# Platelets in Viral Infections – Brave Soldiers or Trojan Horses

**DOI:** 10.3389/fimmu.2022.856713

**Published:** 2022-03-28

**Authors:** Waltraud C. Schrottmaier, Anna Schmuckenschlager, Anita Pirabe, Alice Assinger

**Affiliations:** Institute of Vascular Biology and Thrombosis Research, Centre of Physiology and Pharmacology, Medical University of Vienna, Vienna, Austria

**Keywords:** platelet, platelet activation, infection, virus, viral haemorrhagic fever, influenza, COVID-19

## Abstract

Viral infections are often associated with platelet activation and haemostatic complications. In line, low platelet counts represent a hallmark for poor prognosis in many infectious diseases. The underlying cause of platelet dysfunction in viral infections is multifaceted and complex. While some viruses directly interact with platelets and/or megakaryocytes to modulate their function, also immune and inflammatory responses directly and indirectly favour platelet activation. Platelet activation results in increased platelet consumption and degradation, which contributes to thrombocytopenia in these patients. The role of platelets is often bi-phasic. Initial platelet hyper-activation is followed by a state of platelet exhaustion and/or hypo-responsiveness, which together with low platelet counts promotes bleeding events. Thereby infectious diseases not only increase the thrombotic but also the bleeding risk or both, which represents a most dreaded clinical complication. Treatment options in these patients are limited and new therapeutic strategies are urgently needed to prevent adverse outcome. This review summarizes the current literature on platelet-virus interactions and their impact on viral pathologies and discusses potential intervention strategies. As pandemics and concomitant haemostatic dysregulations will remain a recurrent threat, understanding the role of platelets in viral infections represents a timely and pivotal challenge.

## Platelets and Viruses

While the discovery and availability of antibiotics has dampened the fright of bacterial epidemics, viral infections pose a major risk to global health as currently demonstrated by the COVID-19 pandemic. Viral infections can cause a variety of clinical symptoms upon systemic dissemination, frequently including alterations of the haemostatic system, such as increased risk for thrombosis and/or bleeding.

As cellular mediators of haemostasis platelets are prominently involved in many of these haemostatic disturbances. Owing to their evolutionary heritage, which they share with leukocytes, platelets express receptors for various pathogens, enabling them to directly recognize and respond to invading viruses (1, 2). Thereby, haemostatic platelet functions such as maintenance of vascular integrity or thrombus formation are affected by platelet-virus interactions. Activated endothelial cells and leukocytes also modulate platelet functions during viral infections – either *via* cell-to-cell contacts or indirectly *via* release of circulating mediators. Increasing evidence emerges that platelets themselves function as immune modulators, thereby signalling back to leukocytes and endothelial cells to alter their effector functions (2–5).

As platelets become activated and/or hyper-responsive during viral infections, they modulate other host responses to interfere with infectious pathogens depending on the local environment, the invading pathogen and the disease state. In re-occurring infections platelets further mediate serological memory *via* targeted antiviral IgG release at sites of infection (6).

Their highly sensitive nature in combination with their high abundance therefore renders platelets not just crucial mediators of haemostatic functions but also a formidable first line of defence during viral infections. However, some viruses found ways to exploit platelets as a shelter and transport system through the circulation, turning platelets into a guardian as well as a Trojan horse during viral infections.

## Direct Interactions of Platelets and Viruses

### Molecular Interactions

**Figure 1:**
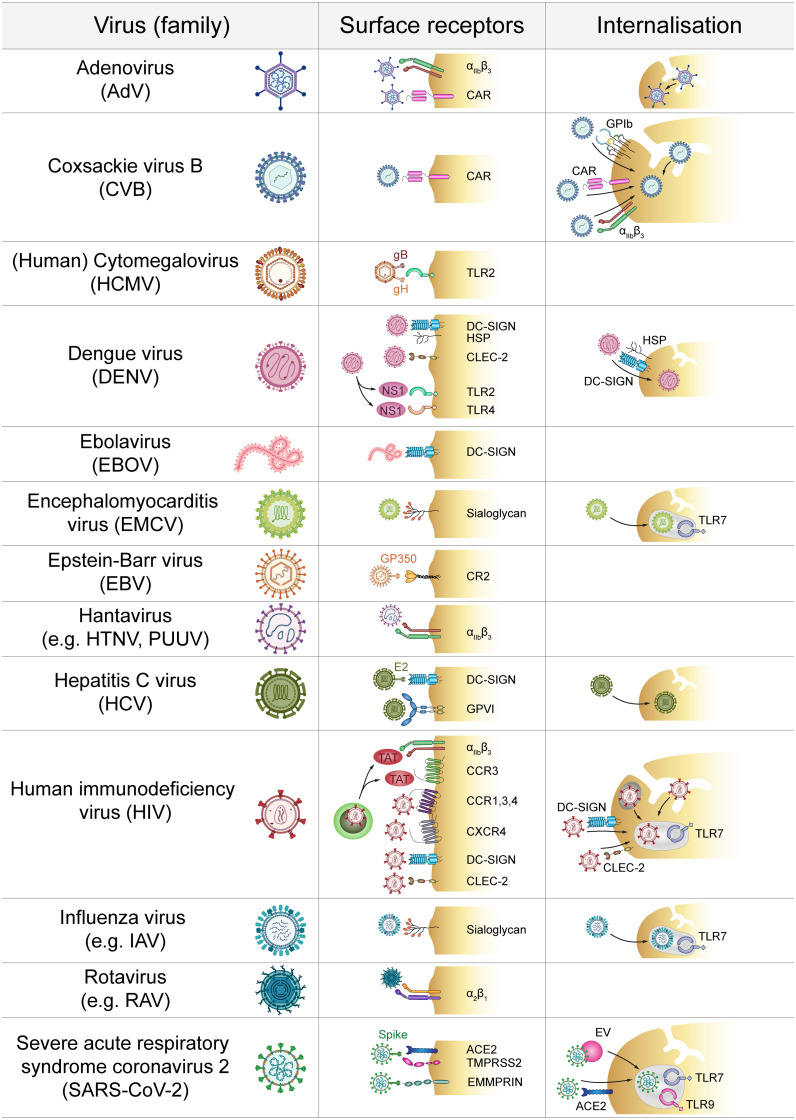
Direct platelet-virus interactions. Platelets express a plethora of surface receptors to directly bind various virus families. Subsequent virus internalisation may occur *via* the cell surface or the open canalicular system. ACE2, Angiotensin-converting enzyme 2; CAR, Coxsackie and Adenovirus receptor; CCR, C-C chemokine receptor; CLEC-2, C-type lectin receptor 2; CR2, Complement receptor 2; CXCR, C-X-C chemokine receptor; DC-SIGN, Dendritic cell-specific intercellular adhesion molecule-3-grabbing non-integrin; GP, Glycoprotein; HSP, Heparan sulfate proteoglycan; HTNV, Hantaan virus; IAV, Influenza A virus; EMMPRIN, extracellular matrix metalloproteinase inducer; EV, Extracellular vesicle; NS1, Non-structural protein 1; PUUV, Puumala virus; RAV, Rotavirus A; TAT, Trans-activator of transcription; TLR, Toll-like receptor; TMPRSS2, Transmembrane protease serine subtype 2.

**Figure 2:**
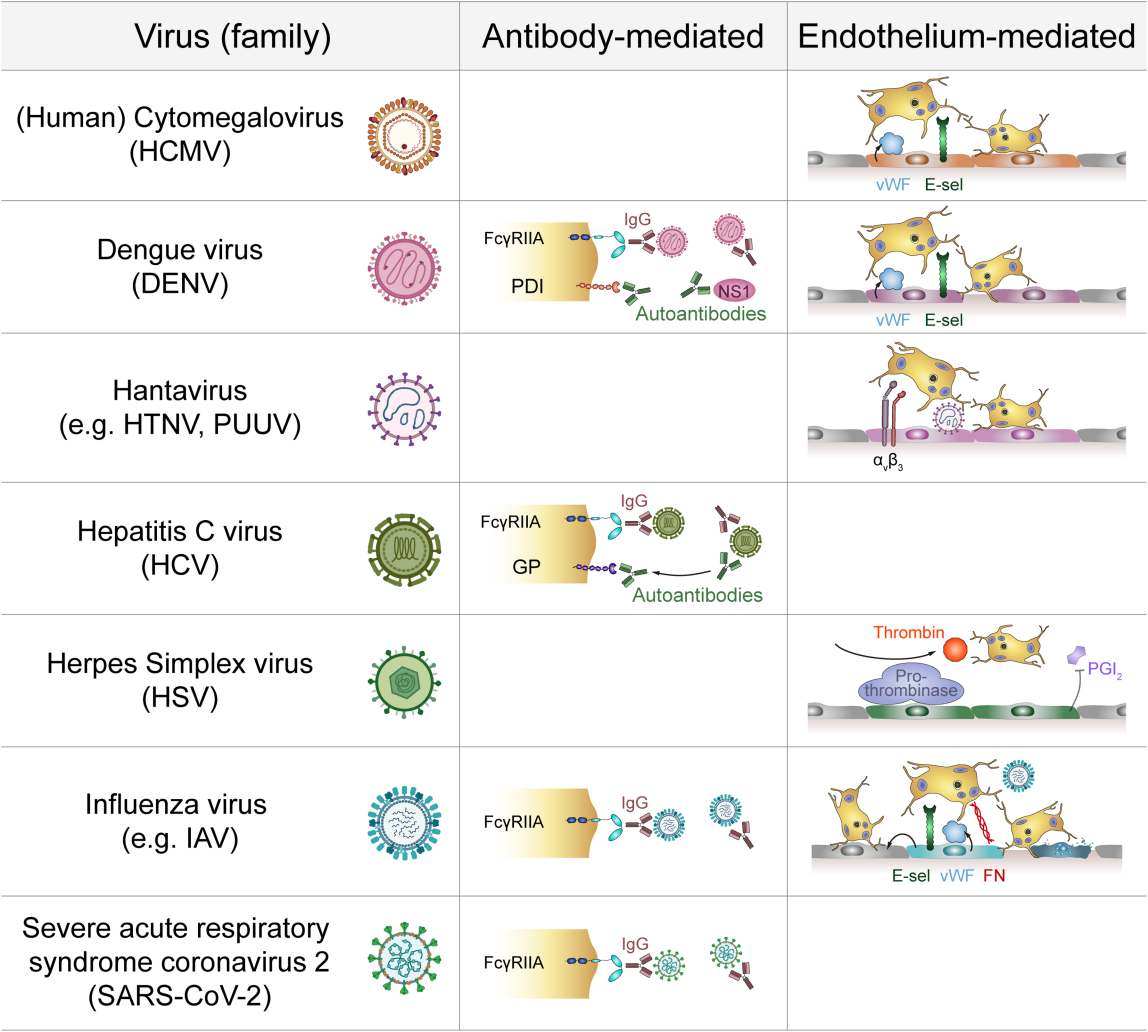
Indirect platelet-virus interactions. Several virus families interact with platelets *via* the formation of virus-IgG complexes that are recognized by platelet FcγRIIA. Antibodies against viral proteins may also act as autoantibodies by targeting platelet membrane components. Additionally, infection of endothelial cells induces expression of adhesive molecules that promote platelet adhesion. Infected endothelial cells also facilitate pro-thrombinase activity to modulate platelet activation. E-sel, E-selectin; FcγRIIA, Immunoglobulin γ Fc region receptor IIA; FN, Fibronectin; GP, Glycoprotein; HTNV, Hantaan virus; IAV, Influenza A virus; IgG, Immunoglobulin G; NS1, Non-structural protein 1; PDI, Protein disulfide isomerase; PGI_2_: Prostaglandin I_2_ (prostacyclin); PUUV, Puumala virus; vWF, von Willebrand factor.

Platelets express various receptors that mediate virus entry into other cell types and direct interactions between platelets and virus were first described in the late 1990s (7, 8). Today we know that platelets express a diverse repertoire of receptors to directly and indirectly interact with viruses (3, 4, 8, 9) which are summarized in **Figures 1** and **2**.

#### Surface Binding

The most rapid interaction between platelets and viruses occurs *via* direct contact with binding and/or entry receptors (**Figure 1**). Platelets express a plethora of receptors that specifically allow for interaction with pathogens (2). However, viruses also use receptors that are required for other physiological functions in order to interact with platelets. In this context integrins are of special importance as they are primarily responsible for platelet adhesion but also bind to and might even facilitate entry of specific virus strains (10). Especially, the abundantly expressed β_3_ integrins are often implicated in binding of viruses, e.g. pathogenic hantaviruses (11). Adenovirus-platelet interaction also involves β_3_ integrins, such as α_IIb_β_3_ and α_V_β_3_ (12–14), as blocking prominent β_3_ integrins does not completely abolish virus internalization (15). Other integrins such as α_2_β_1_ are involved in viral binding e.g. of rotaviruses (16).

Further, platelets express the Coxsackie and Adenovirus receptor (CAR) (12, 17) which allows for interaction between platelets and Coxsackie virus B (CVB) (18), as well as other viruses (19).

Sialic acid acts as a cellular receptor which interacts with heavily glycosylated glycoproteins (20). As platelets express sialic acid on their surface (21) these sialoyglycans enable interaction with various viruses such as Encephalomyocarditis virus (EMCV) (22) and Influenza virus (23–25).

Also, various cytokine receptors are hijacked by viruses to mediate their interactions with platelets. In that regard, Human immunodeficiency virus (HIV) illustrates how diverse virus-platelet interactions can be. *Via* expression of C-X-C chemokine receptor (CXCR) type 4 and the required co-receptors C-C chemokine receptor (CCR) type 1, 3 and 4 platelets can directly interact with HIV particles (26, 27). Also, HIV infected cells release the protein Trans-Activator of Transcription (TAT), which activates platelets by binding concurrently to integrin β_3_ and CCR3 (28). Platelets also bind and internalize HIV *via* dendritic cell-specific intercellular adhesion molecule-3-grabbing non-integrin (DC-SIGN) (29–31) and C-type lectin receptor 2 (CLEC-2) (30). DC-SIGN further binds to Ebola virus (EV) (32) which is most likely only captured (33). Hepatitis C virus (HCV) also binds DC-SIGN *via* viral glycoprotein E2 (34) but also to the collagen receptor glycoprotein (GP) VI (35). For Dengue virus (DENV) a dual receptor recognition involving DC-SIGN and heparan sulfate proteoglycan (HSP) has been suggested to mediate primary platelet binding (36–38). As platelets express no efficient entry receptor, it is not yet fully understood whether binding to DC-SIGN simply mediates virus capture or whether this interaction is also sufficient for cellular entry of HIV and HCV (31, 34).

Apart from DC-SIGN, DENV induced platelet activation also involves CLEC-2 (39). Moreover, DENV non-structural protein 1 (NS1) interacts with Toll-like receptor (TLR) 4 and TLR2, but only TLR4 also mediates platelet activation (40, 41). TLR2 is also important for platelet interaction with Human Cytomegalovirus (HCMV) (42) probably *via* HCMV glycoproteins B and H (43). Platelets also express complement receptors (CRs) on their surface which allows for the binding of Epstein-Barr virus (EBV) glycoprotein GP350 to platelet CR2 (44, 45).

The interaction of platelets with SARS-CoV-2 is rather controversial. Angiotensin-converting enzyme 2 (ACE2) is proposed as an entry receptor for SARS-CoV-2, which directly interacts with the spike protein (46). However, while some reports demonstrate ACE2 expression on platelets (47), other studies did not find detectable level of ACE2 but suggest mechanisms independent of ACE2 (48), while others found no virus particles within platelets at all (49). Given the low expression of ACE2 on platelets, ACE2-independent platelet activation involving more abundantly expressed receptors such as extracellular matrix metalloproteinase inducer (EMMPRIN/CD147) may be more important for direct platelet dysregulation by SARS-CoV-2 or its spike protein, respectively (50).

#### Internalization of Virus

Platelets can not only bind but also take up virus particles. However, whether this uptake resembles a true phagocytosis, simple engulfment of virus or a different mechanism remains to be elucidated and might depend on the type of virus and receptors involved. Internalisation of HCV increases virus persistence by sheltering virus from degradation (51–54). DENV particles can be bound and internalized *via* DC-SIGN and HSP (37). Accordingly, DENV virions were also detected within platelets of infected patients and the presence of negative stranded RNA upon virus uptake suggest that platelets are permissive for virus replication, but this probably does not result in productive infection (55, 56). Other viruses, such as CVB and adenovirus were detected in the surface-connected open canalicular system (OCS) (15, 57), which is involved in internalisation processes. While uptake of CVB is mainly mediated by CAR, the OCS, platelet integrin α_IIb_β_3_ and GPIb might also be involved (19).

Similarly, HIV particles can be engulfed in the OCS but also in vacuoles close to the plasma membrane. Some of these vacuoles resemble or are enclosed by endosome-like structures, suggesting phagocytosis along with simple uptake (27, 58, 59). However, the precise mechanism has not yet been unravelled and might involve HIV co-receptors along with yet unidentified mediators (27, 30). Recently, internalised HIV virions were found to be shuttled from early to late endosomes in an endocytic machinery-dependent manner, providing evidence of true phagocytosis by platelets (29, 59).

Internalisation of viral particles by platelets allows for stimulation of intracellular pattern recognition receptors such as TLRs, which has been observed for HIV and platelet TLR7 (60), but also EMCV and influenza virus are internalised by platelets (61) and their uptake involves TLR7 activation (62). This might represent a general mechanism how single-stranded RNA viruses interact with platelets and subsequently influence their activation. As yet another uptake mechanisms, successful internalisation of SARS-CoV-2 by platelets may be independent of ACE2 expression as virions were found to hitchhike on extracellular vesicles that fuse with the platelet membrane (63).

### Effects

**Figure 3:**
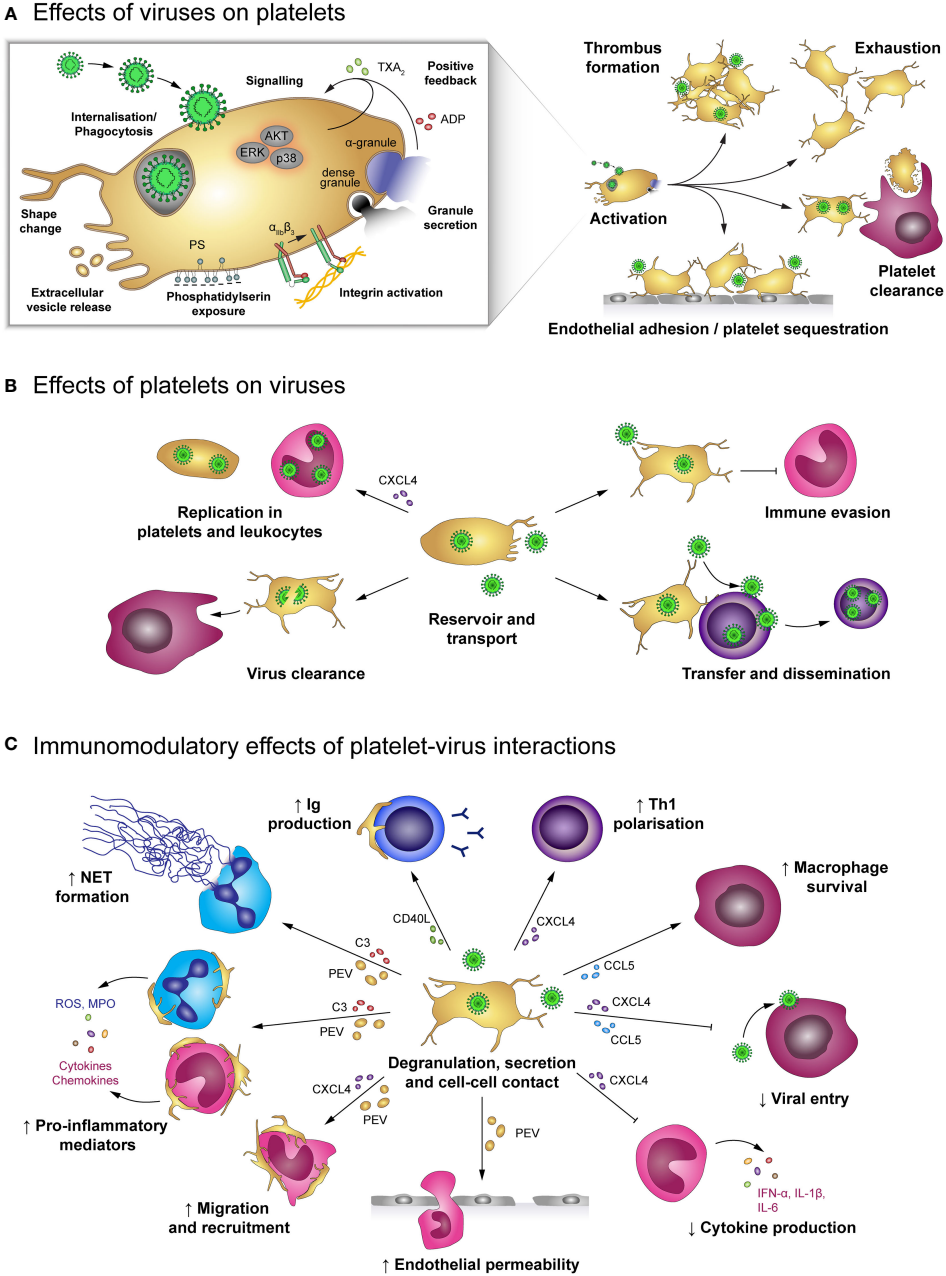
Effects of platelet-virus interaction. **(A)** Virus binding or internalisation/phagocytosis stimulates platelet activation including degranulation, integrin activation, phosphatidylserin exposure and extracellular vesicle release. Virus-induced platelet activation enhances endothelial adhesion and thrombus formation, but may also lead to platelet exhaustion and clearance from the circulation. **(B) **Platelets serve as virus reservoir and transport vehicle by supporting viral replication in leukocytes or within platelets themselves and by transferring virus to other host cells, thereby augmenting viral dissemination. Furthermore, platelets shelter intracellular virions from immune-mediated degradation, though degradation of virus-containing platelets contributes to virus clearance. **(C) **Virus-stimulated platelets modulate immune responses by cell-cell contact and/or soluble mediators. Platelet-derived factors block viral entry into leukocytes and prolong macrophage survival. Platelets also facilitate leukocyte extravasation by augmenting endothelial permeability, leukocyte migration and recruitment. Furthermore, platelets regulate leukocyte effector functions such as IgG production, T cell polarization, NET formation and release of inflammatory mediators, thereby either enhancing or diminishing immune defences. ADP, adenosine diphosphate; AKT, Protein kinase B; C3, complement factor 3; CCL5, chemokine (C-C motif) ligand 5; CD40L, CD40 ligand; CXCL4, chemokine (C-X-C motif) ligand 4; ERK, Extracellular signal-regulated kinase; IFN-α: Interferon α; IgG, immunoglobulin; IL-1β: Interleukin 1β; MPO, myeloperoxidase; NET, neutrophil extracellular trap; p38, p38 mitogen-activated protein kinase; PEV, Platelet-derived extracellular vesicle; PS, phosphatidylserine; ROS, reactive oxygen species; Th1, T helper cell type 1; TXA_2_, thromboxane A_2_.

Platelets not only express several receptors for the recognition of pathogens but are also equipped with a plethora of antimicrobial molecules to fight viral infections. Interaction between platelets and viruses can thus have benefits and risks for the host: while direct interactions can lead to phagocytosis and degradation of viral particles, platelets can also become a hideout for viruses, thereby fostering reproduction and dissemination (**Figure 3**). Apart from their role in haemostasis, platelets represent relevant regulators of immune responses, e.g. by interacting with leukocytes and subsequently modulating their extravasation and effector functions (5, 64, 65). Thereby, quantification of circulating platelet-leukocyte aggregates (PLAs) is often used to gain insight into immunomodulatory platelet function.

#### Platelet Activation in Response to Viruses

Direct interaction of viruses and platelets is associated with platelet activation indicated by elevated P-selectin (36, 37, 39, 42, 66–68), CD63 (39) and CD40L expression (67), α_IIb_β_3_ activation (66, 68), phosphatidylserine (PS) exposure (36, 68) as well as increased secretion of platelet-derived extracellular vesicles (PEV) (39, 66, 68), platelet factor 4 (PF4/CXCL4) (69), Thromboxane A2 (TXA_2_) (66) and ADP/ATP (42). In addition, enhanced AKT, p38 and ERK phosphorylation (42) and changes in the platelet morphology (67) support the notion that platelets are stimulated by the direct interaction with viruses (44, 55, 59, 67, 70). As a consequence, platelet activation promotes endothelial adhesion (39) and coagulation (44, 69, 70).

#### Viral Uptake, Degradation and Replication

Despite their seemingly simple nature, platelets have been known for decades to bear the ability to kill viral particles by every trick in the book of phagocytosis, including attachment, invagination and formation of phagosome-like structures (7). Viruses are ensnared in endocytic vesicles and killed by antimicrobial peptides when the vesicles fuse with granules. For HIV, this uptake occurs mainly in the OCS, where viruses lose their regular morphology (69) and envelope (29, 59), while phagocytosis of influenza virus is OCS independent (62, 71).

Studies on patient-derived blood showed that platelets rapidly internalise and digest influenza A (H1N1 or H3N2) by fusion of vesicles and granules, suggesting that during viremic influenza infection platelets contribute to viral killing (62, 71). Findings on platelet interaction with HIV are still controversial. While some reports indicate HIV degradation in platelets, others found that platelets can take up HIV particles and keep them in an infectious state. Infectious HIV can then be transmitted from platelets to T cells or dendritic cells, which facilitates viral dissemination (30). This is of special importance, as platelets from HIV-infected individuals on combined antiretroviral drug therapy with low blood CD4+ T cell counts contain replication-competent HIV despite viral suppression (72). Dengue virus particles were also observed in vesicles of patient-derived platelets and Dengue structural protein E was detected inside platelets or in platelet micro-aggregates of *in vitro* infected platelets (56). However, it is currently unclear whether platelets degrade dengue virions or if the virus enters and/or infects platelets to evade immune responses. While it is clear that platelets directly interact with HCV (51, 73, 74), little is known on internalisation and or degradation of HCV by platelets. But the fact that even after anti-viral therapy HCV RNA was undetectable in serum but still detectable in platelets suggests that platelets serve as a reservoir for HCV and protect them from immune recognition (73).

Intriguingly, although being an anucleated cell, platelets may also serve as host cells for virus replication. Indeed, platelets with internalised Dengue virus particles were shown to enable replication of the viral genome (+ssRNA) of all Dengue virus serotypes (37). During acute infection a high number of Dengue associated copy numbers could be found in Dengue patients’ platelets (68) and isolated platelets contain a higher proportion of Dengue virus-associated RNA than plasma (56), suggesting that Dengue virus infects platelets for reproduction (37, 68). However, while Dengue virus reproduction in platelets themselves is rather inefficient (55), Dengue virus activated platelets enhance viral replication in monocytes and THP-1 cell lines *in vitro via* secretion of PF4/CXCL4 (75).

#### Platelet Mediated Immune Responses

Platelet activation in response to viruses can thus indirectly contribute to antiviral effects *via* modulation of immune responses.

Platelet-virus interaction triggers the release of cytokines and chemokines from platelets including tumour necrosis factor α (TNF-α), interleukin (IL) 6, IL-8, IL-10, monocyte chemoattractant protein 1 (MCP-1/CCL2), transforming growth factor β (TGF-β) and the granulocyte-macrophage colony stimulating factor (GM-CSF) (67). These mediators are important for the initiation of cell migration and immune defence mechanism. In addition, formation of platelet-leukocyte and platelet-lymphocyte aggregates facilitates leukocyte activation, recruitment and reactive oxygen species (ROS) formation (42) as well as B cell isotype switching to immunoglobulin (Ig) G production (76).

In addition to direct platelet-leukocyte interaction, Dengue virus-stimulated platelets also release PEV that enhance vascular permeability, thereby promoting leukocyte migration, and also stimulate the release of pro-inflammatory cytokines such as TNF-α and IL-6 by neutrophils and macrophages. Furthermore, virus-stimulated platelets play a role in formation of neutrophil extracellular traps (NETs) (39), the release of neutrophil-DNA and myeloperoxidase (MPO), which is provoked by platelet-derived complement factor C3 (62).

However, platelet-virus interaction can also have immunosuppressive functions. In acute and chronic EBV infections platelet-derived TGF-β inhibits lung epithelial growth and contributes to disease pathology (44). Also, PF4/CXCL4 diminishes the immune defence against Dengue virus *in vitro* by inhibiting monocyte secretion of interferon α (IFN-α), IL-1β and IL-6 (75). In addition, activated platelets may influence antiviral immune defences by modulating infection of leukocytes themselves. PF4/CXCL4 reduces HIV infection of T cells and macrophages by binding a subunit of the viral envelope glycoprotein 120 (gp120) and thus blocking viral entry (69, 77, 78).

Dengue patients often present with upregulated platelet apoptosis, indicated by mitochondrial depolarization, elevated PS exposure and high expression of caspase-3 and caspase-9 (36, 79). Moreover, increased platelet levels of annexin V, cyclophilin D and thrombopoietin (TPO) are associated with diminished platelet count as apoptotic platelets get phagocytosed by monocytes *via* PS recognition (68, 79). Similarly, platelets of dengue patients have impaired mitochondrial membrane potential and constitutively generate ROS, indicating that platelet mitochondria are impaired in viral infections (36).

#### Thrombocytopenia

Thrombocytopenia is a hallmark of viral infections and the underlying causes are multifaceted and complex (2). Thrombocytopenia can be induced by four major mechanisms: (1) direct destruction of circulating platelets, (2) platelet destruction by immunological features such as auto-antibodies, (3) dysregulation of platelet production (megakaryopoiesis and thrombopoiesis) or (4) direct destruction of megakaryocytes (36).

Platelet counts often inversely correlate with viral loads and disease progression, indicating a hallmark in disease pathology, though the underlying mechanisms vary (70, 73, 78). Dengue-associated thrombocytopenia is related to complement-antibody mediated clearance and lysis of activated platelets (36, 37, 68, 75), while platelet activation contributes to thrombocytopenia in HCV infection (73). Influenza-associated thrombocytopenia is, dependent on the influenza strain, induced by viral neuraminidases, which remove sialoglycans after internalisation, thereby stimulating platelet clearance by liver hepatocytes and macrophages (7, 70). In contrast, *in vitro* Dengue and Junin virus can infect hematopoietic progenitors, which induces production of type I IFN, which in turn attenuates pro-platelet production (80, 81). Interestingly, upregulation of antiviral immune genes e.g. interferon-induced transmembrane protein 3 (IFITM3) in megakaryocytes in response to Dengue infection prevents infection of neighbouring megakaryocytes (81). Of note, patients with an early and sustained anti-viral response may have lower platelet counts during acute infection than patients with no-early response (73). Severe systemic viral infections often result in disseminated intravascular coagulation (DIC), in which excessive and systemic activation of the coagulation system leads to increased consumption of coagulation factors and platelet activation and ultimately results in formation of microthrombi. The subsequent drop in platelet count and concurrent haemorrhages represent a most dreaded clinical complication (82).

## Indirect Interactions in Viral Diseases – Platelets in the Inflamed Environment

Indirect platelet-virus crosstalk on the molecular level may involve additional factors that bridge the contact between platelet receptors and virus particles, but indirect interactions may also be mediated on cellular level when platelets communicate with virus-infected or –activated cells (**Figure 2**).

Platelets also express FcγRIIA, which is a low-affinity receptor for the constant domain of IgGs. This allows for binding of virus-containing immune complexes that are generated during viral infections (83) and contribute to viral pathogenicity (66). As binding of immune complexes to FcγRIIA activates platelets (66, 84), indirect binding of virions to platelets *via* bridging IgGs may stimulate platelets even in the absence of virus-specific receptors. Indirect binding of virus-containing immune complexes to platelet FcγRIIA has been described for dengue virus, influenza virus and HCV (35, 68, 85).

Apart from virus-IgG complexes also autoantibodies are generated during infections which are recognized by FcγRIIA. In HCV infection autoantibodies that are cross-reactive towards platelet glycoproteins have been described (86). Autoantibodies against platelets are also common during DENV infection (87, 88). Here, antibodies which initially target DENV NS1 protein are cross-reactive with protein disulfide isomerase (PDI) expressed on the platelet surface (89).

Platelets are primarily known for their role in haemostasis and as such are pivotally involved in preventing and stopping haemorrhages by attaching to the injured endothelium and sealing vessel gaps. This interaction is mediated by various integrins and surface receptors such as α_IIb_β_3_, GPIbα and P-selectin (90), which are also involved in viral interactions. Infected endothelial cells thus also contribute to platelet activation by upregulating adhesion molecules, which influence the binding and interaction with platelets (91). For example, infection with various Hantaviruses e.g. Andes or Hantaan virus induces upregulation of endothelial β_3_ integrins, which are involved in the recruitment of platelets (11). However, β_3_ also displays the virus on the endothelial surface, which results in platelet recruitment, as shown for Sin Nombre virus (92). Platelets also bind to endothelial cells infected with Puumala hantavirus (PUUV) (93), which may contribute to the low platelet count observed in patients with PUUV infection.

Increased platelet-endothelium interactions are also observed in DENV (94), Influenza virus (95) and HCMV infection (96). DENV infected endothelial cells express increased E-selectin levels, which in turn promotes platelet adhesion and activation (94). Moreover, DENV-derived NS1 protein triggers degranulation of Weibel-Palade bodies, leading to increased secretion of von Willebrand Factor (vWF) (40), further augmenting platelet adhesion and activation. Similarly, active HCMV replication in endothelial cells increases their expression of vWF and intercellular adhesion molecule 1 (ICAM-1), which mediates platelet-endothelial interactions (97) *via* platelet GPIb (96). Although vWF and ICAM-1 are upregulated during influenza infection, platelet adhesion to influenza-infected endothelial cells is primarily mediated by other molecules, such as endothelial fibronectin and platelet α_5_β_1_. Moreover, paracrine mechanisms also contribute to platelet adhesion in influenza infection, as platelets also attach to cells neighbouring infected ones (95).

In addition to triggering endothelial expression of pro-thrombotic factors and adhesion molecules, virus infection of endothelial cells may also promote endothelial permeability, thereby causing exposure of the subendothelial matrix and subsequent platelet adhesion. Indeed, interaction of DENV with TLR4 was shown to disrupt endothelial integrity (98) and infection of pulmonary microvascular endothelial cells with Influenza virus increases endothelial permeability by inducing apoptosis (99).

Regulation of platelet binding to infected endothelium may have clinical significance. As such, *in vitro* endothelial cells infected with herpes simplex virus (HSV) display changes in surface composition that facilitate the assembly of the pro-thrombinase complex and thereby accelerate thrombin generation, while at the same time prostacyclin (prostaglandin I_2_; PGI_2_) secretion is impaired. Together, these changes in endothelial activation promote platelet binding and induce a pro-thrombotic and pro-coagulant microenvironment (100) that may foster thromboembolic complication.

## Contribution of Platelets to Viral Pathologies

Platelet interaction with viruses contributes to the pathologies of a plethora of infectious diseases. In the following section we focus on three types of viruses, which are associated with haemostatic imbalances. We summarize the current knowledge on viral haemorrhagic fever (VHF), an infectious disease associated with an increased bleeding risk, influenza and COVID-19, which are primarily pulmonary diseases but also associated with thrombotic complications.

### Haemorrhagic Viruses

**Figure 4:**
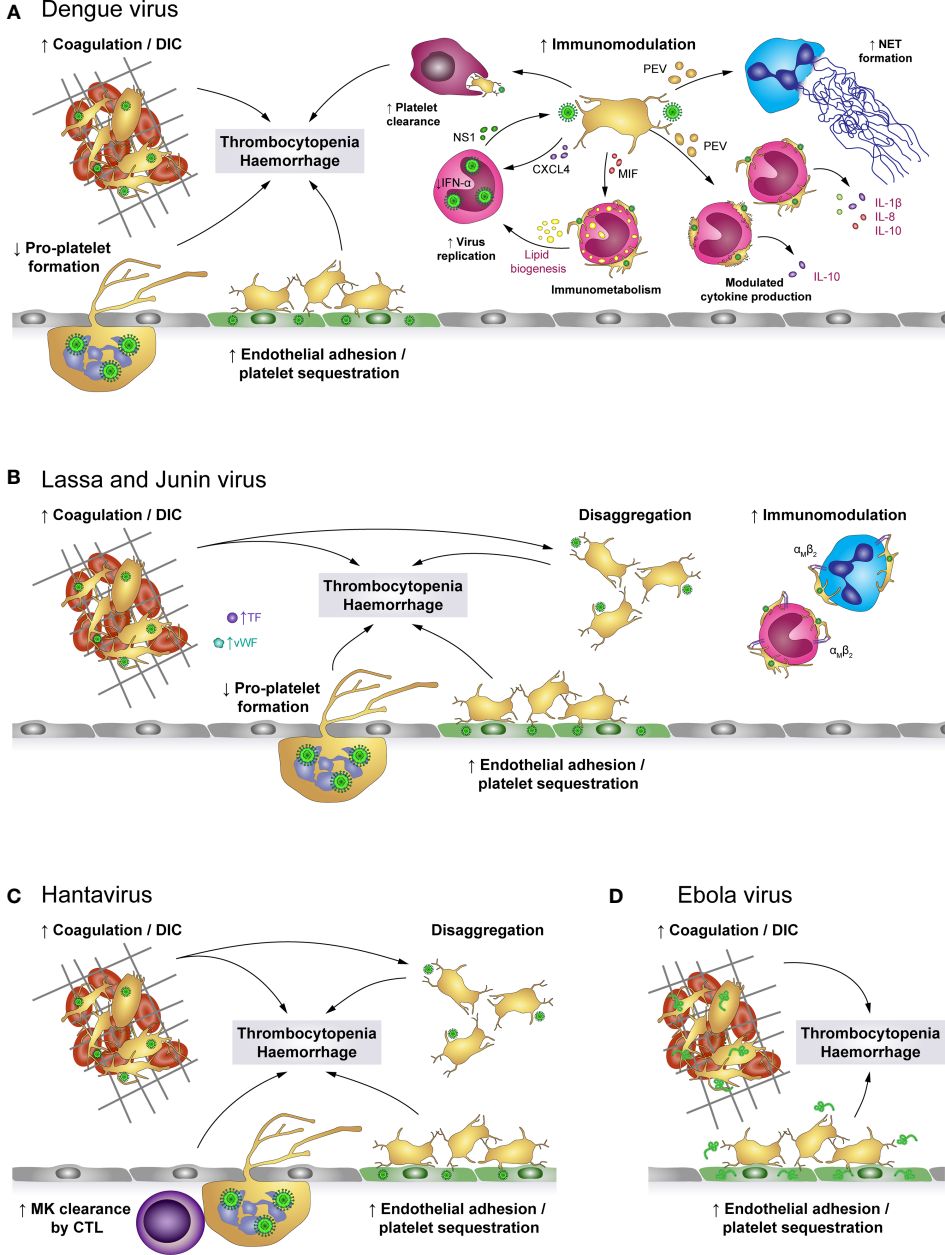
The role of platelets in viral haemorrhagic fevers (VHF). **(A)** Dengue virus activates platelets and promotes the development of disseminated intravascular coagulation (DIC), leading to platelet consumption. Additionally, activated platelets adhere to infected endothelial cells and infected megakaryocytes (MK) show impaired pro-platelet formation. Together with macrophage-mediated clearance of virus-activated platelets, these mechanisms lead to thrombocytopenia and enhanced haemorrhagic complications. In addition, virus-stimulated platelets bind to leukocytes or release soluble factors to modulate immune responses such as NET formation as well as production of a specific cytokine profile, depending on whether interacting platelets are activated or apoptotic. Platelet-derived factors also promote virus replication in monocytes which is further fostered by modulated immunometabolism and platelet-induced lipid biogenesis. **(B)** Infection with Lassa or Junin virus is associated with elevated levels of von Willebrand factor (vWF) and tissue factor (TF) which contribute to platelet activation and development of DIC. While activated platelets readily bind to monocytes and neutrophils *via* their α_M_β_2_ receptor, they are unable to maintain stable platelet-platelet interaction and rapidly disaggregate. Further, virus infection augments endothelial platelet adhesion and sequestration and limits pro-platelet formation of MKs. Thereby, infection with Lassa or Junin virus leads to thrombocytopenia and increases bleeding risk. **(C)** Hantavirus readily infects endothelial cells which promotes endothelial platelet adhesion and sequestration from the circulation. While infected megakaryocytes display no differentiation dysfunction, they are cleared by cytotoxic T lymphocytes (CTL). Systemic infection triggers a pro-coagulatory state which may exacerbate to DIC. Nevertheless, platelets show impaired capacity to form stable aggregates. Hantavirus thus causes thrombocytopenia and haemorrhagic complications by affecting key mediators of haemostasis. **(D)** Infection with Ebola virus is associated with systemic coagulation induction and risk for DIC, which leads to platelet consumption. However, platelets may also be sequestrated due to enhanced adhesion to the infected endothelium. Thereby, Ebola virus causes thrombocytopenia and haemorrhagic complications. CTL, Cytotoxic T cell; CXCL4, chemokine (C-X-C motif) ligand 4; DIC, Disseminated intravascular coagulation; IFN-α: Interferon α; IL, Interleukin; MIF, Macrophage migration inhibitory factor; MK, Megakaryocytes; NET, Neutrophil extracellular trap; NS1, Non-structural protein 1; PEV, Platelet-derived extracellular vesicle; TF, Tissue factor; VHF, Viral haemorrhagic fevers; vWF, von Willebrand factor.

The term viral haemorrhagic fever (VHF) describes an infectious disease associated with an increased bleeding risk. VHF is caused by a distinct group of enveloped RNA viruses that belong to four virus families: Flavivirus (Dengue virus, Yellow fever virus), Bunyaviridae (Hantavirus, Crimean-Congo haemorrhagic fever virus), Arenaviridae (Lassa virus, Junin virus) and Filoviridae (Ebola, Marburg and Sudan virus). An infection with these viruses causes systemic pathogenesis characterized by fever, malaise but also increased vascular permeability and the development of thrombocytopenia, both supporting increased bleeding tendencies (**Figure 4**). These vascular and haemostatic dysregulations often lead to coagulopathies, which aggravate disease outcome. VHFs are associated with high mortality rates. However, currently no effective therapeutic interventions are known, making them a major global health problem (101). Although, the underlying molecular pathways and mechanism are poorly understood, an involvement of platelets as main players of haemostasis is likely (102).

Various mechanisms were shown to trigger thrombocytopenia in VHFs (102), though depending on the virus strain different mechanisms dominate. Especially, virus-induced tissue damage and DIC result in excessive platelet activation and hence consumption. DIC is observed during DENV infection (103–105), in Ebola (106, 107) and also in Haemorrhagic Fever with Renal Syndrome (HFRS) caused by PUUV (108). Accordingly, thrombocytopenia along with abnormal coagulation is a known hallmark of pathogenic Hantavirus infection (109, 110), while in Lassa fever DIC seems to play only a minor role (111–113).

Also endothelial cells contribute to haemostatic dysregulations in VHFs. On the one hand infection of endothelial cells induces tissue factor expression, leading to increased thrombin generation and thereby haemostatic disturbances, which increase the risk for clot formation (93). Accordingly, D-dimer levels and prothrombin fragments F1 + 2, are increased in Dengue patients (93), patients with fatal Crimean Congo Haemorrhagic fever (114) and Ebola (106).

On the other hand the infected endothelium contributes to diminished platelet counts *via* scavenging and/or activation of circulating platelets. Platelet adhesion to DENV-infected endothelium is partially prompted by increased expression of endothelial E-selectin and P-selectin, which further contributes to a drop in circulating platelets (94). Also Ebola patients show elevated E- and P-selectin levels, indicating endothelial cell and platelet activation, which might contribute to diminished circulating platelets due to endothelial scavenging (115). Platelet adhesion to endothelial cells was further observed during PUUV (93) and Hantaan virus (HTNV) infection (11).

DENV-derived NS1 also directly increases platelet adhesion. NS1 activates platelets *via* TLR-4, thus supporting increased endothelial adhesion but also enhancing platelet aggregation (40). Subsequently, NS1 activated platelets are prompted to perform a phenotypic switch towards inflammation including degranulation, the synthesis but not the release of IL-1β as well as the continued expression and release of NS1 after infection. In turn, released NS1 and granule-stored factors might further enhance platelet activation and aggregation in an autocrine loop (41). Enhanced platelet activation is additionally triggered by DENV-induced synthesis and expression of human leukocyte antigen (HLA) class 1 on platelets which then binds circulating cell-free H2A histones, found in dengue patients’ plasma (116). Enhanced platelet activation and adhesion thus represent a possible link to thrombocytopenia and haemorrhages in dengue fever patients.

Intriguingly, in Lassa fever patients with fatal outcome and during the acute phase of Hantavirus infection dysfunctional platelets with an impaired aggregation potential were found (117). Indeed, normal aggregation is rapidly followed by disaggregation, indicating that Lassa virus (LASV) infection triggers an aggregation inhibition, which is likely due to impaired platelet degranulation as degranulation is essential for sustained platelet aggregation (111, 118, 119). Similarly, infection with Junin virus (JUNV), another virus belonging to the family of Arenaviridae causing Argentine Haemorrhagic Fever, is associated with decreased platelet aggregation, which is mediated by an unidentified plasma component present during acute infection (120). As a consequence, diminished platelet aggregation might contribute to bleeding events that frequently occur in these patients.

Additionally, several proteins important for haemostasis such as TF and vWF are also increased in plasma from Lassa patients, which further implicates pathogenic activation of the coagulation system as well as platelets (111). Similarly, patients suffering from haemorrhagic manifestations during Sudan virus infection also show elevated levels of vWF. This further implicates that VHF viruses contribute to excessive thrombotic events (121).

Phagocytosis of platelets is another mechanism leading to a decrease in circulating platelets. Platelets are cleared *via* phagocytosis by macrophages upon activation by DENV (40) or severe fever with thrombocytopenia syndrome virus (SFTSV), a member of the Bunyaviridae family like Hantavirus (122). Platelets from dengue patients further shows decreased sialic acid levels, which are associated with increased platelet phagocytosis and hence platelet clearance (123, 124). In line, Dengue fever is associated with increased levels of circulating active vWF, which also induces removal of sialic acids on platelets *via* neuraminidase mobilization (124). These observations suggest that the underlying mechanisms of thrombocytopenia include platelet clearance.

Not only dysfunctional platelets and/or reduced platelet counts are problematic in VHF, but additional activation of the immune systems e.g. by platelets can further exacerbate disease progression. Platelets also have immunomodulatory functions during VHF. Leukocyte integrin α_M_ (ITGAM, CD11b) is upregulated upon exposure to LASV, which may enhance platelet-leukocyte binding and therefore complicate symptoms (125, 126). During EV infection, a transient increase in sCD40L was measured during the early stages of infection which might be implicated in activating further immune cells (107).

In DENV infection, activated platelets release PMVs, which induce neutrophil activation and subsequent NET formation (39). Additionally, DENV-exposed platelets can also reprogram immunometabolism of uninfected monocytes and amplify potential inflammatory cytokine release. Moreover, by forming aggregates with platelets, monocytes become activated and increase lipid droplet (LD) biogenesis (127). LDs play an important role in the pathogenesis of DENV infection. Viruses are unable to perform lipid synthesis which is a pre-requisite for functional virion production (128) and must therefore exploit cellular mechanisms (129). Indeed, leukocytes from Dengue patients show high LD formation, suggesting that lipid biogenesis might contribute to Dengue pathogenesis (129, 130).

Platelets from dengue patients show increased PS exposure, depolarization of mitochondria as well as the activation of caspase 9 and caspase 3. Thereby DENV-induced platelet apoptosis is not only associated with increased thrombocytopenia but may also enhance monocyte binding (36). Indeed, DENV-activated and apoptotic platelets form aggregates with monocytes and induce secretion of IL-1β, IL-8 and IL-10 or only IL-10, respectively, in a mechanism involving P-selectin-mediated binding as well as recognition of exposed PS (131). All these mechanisms suggest that DENV actually induces cellular changes on many different levels by triggering platelet activation which might aggravate the disease (39).

Lastly, platelets induce the inhibition of IFN-α production in monocytes and enhance DENV replication in monocytes. This effect is at least partially dependent on PF4/CXCL4 and increased plasma levels of PF4/CXCL4 correlate with increased DENV NS1 in monocytes from patients (75).

However, not only platelets but all their progenitors, including both megakaryocytes and haematopoietic stem cells, are affected by VHFs. DENV is able to infect and replicate in megakaryocytes, which leads to diminished megakaryocyte development and platelet production due to diminished pro-platelet formation (132, 133). Similarly, JUNV infection of megakaryocytes also impairs thrombopoiesis by decreasing pro-platelet formation. Reduced pro-platelet formation together with abrogated aggregation responses of circulating platelets contribute to haemorrhages in patients with JUNV infection (80). Also HTNV infects megakaryocytes (134), however no direct effects on megakaryocyte differentiation and survival were observed. Nevertheless, infected megakaryocytes are cleared by cytotoxic T cells, thus also supporting thrombocytopenia (135).

### Influenza

Influenza is an acute respiratory infection caused by negative-strand RNA viruses belonging to the Orthomyxoviridae family that cause seasonal influenza pandemics. While three distinct types of influenza viruses (A, B and C) can infect humans (136, 137), influenza C virus mainly infects children, causing only mild upper respiratory tract infections. Therefore available vaccines target influenza A and B viruses, but not influenza C (138–140). Especially influenza A viruses regularly acquire adaptive mutations by genetic drifts and shifts that create novel influenza A subtypes (136). The virion envelope of influenza is covered with hemagglutinin (HA) and neuraminidase (NA) glycoproteins, which determine the specificity of the virus for a host species and cell type. Each Influenza A subtype is characterized by numbering of HA and NA proteins. The two subtypes H1N1 and H3N2 currently circulate in the human population (139). In contrast, Influenza B virus has only a single subtype with two lineages and is not further sub-categorized (139, 141).

**Figure 5:**
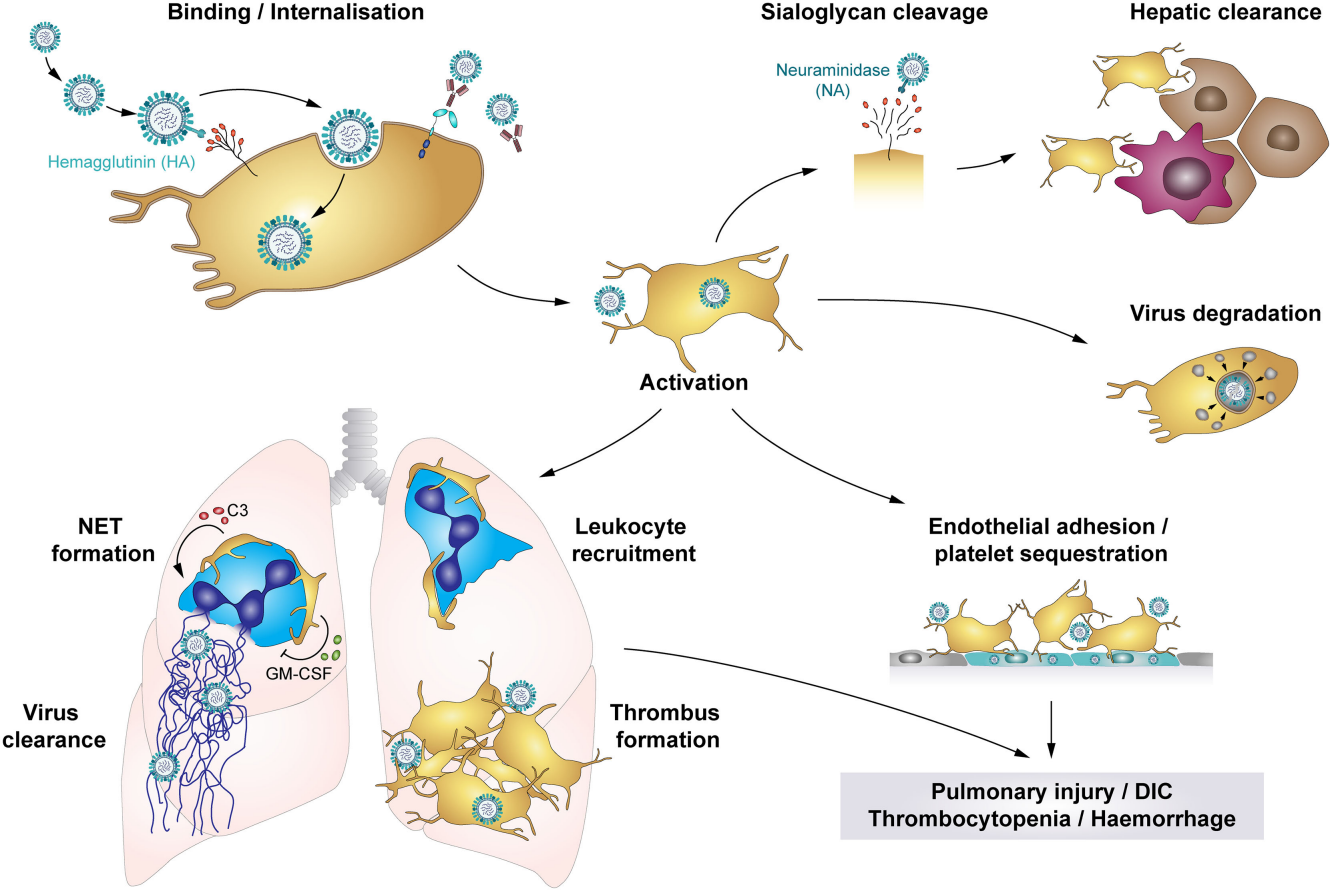
The role of platelets in influenza infections: Platelets bind and internalise influenza virus *via* the interaction of virus hemagglutinin (HA) proteins and sialic acid (SA)-terminated glycans on the platelet surface, though platelets also bind influenza-containing immune complexes. These interactions result in platelet activation and platelet-mediated immune responses that contribute to influenza-associated pathologies. Cleavage of SA residues by viral neuraminidase (NA) induces platelet clearance by liver hepatocytes and Kupffer cells. However, rapid internalisation of viral particles leads to digestion of influenza virus when vesicles with enclosed virions fuse with platelet granules that contain antimicrobial peptides. Systemically, influenza-infected endothelial cells express pro-thrombotic factors like fibronectin or integrin α_5_β_1_, which increases platelet adhesion to the endothelium and thus fosters platelet sequestration. Locally, platelets infiltrate the lung tissue, where thrombus formation may constrict and/or occlude blood vessels or small airways. Furthermore, platelet-leukocyte aggregate formation induces leukocyte recruitment and triggers the formation of NETs to entrap viral particles, regulated by C3 and GM-CSF. However, NETs cause further tissue damage and enhance thrombus formation. Together, these platelet-mediated responses in influenza trigger pulmonary injuries, disseminated intravascular coagulation (DIC), thrombocytopenia and haemorrhages in severe influenza infections. C3, Complement factor C3; DIC, disseminated intravascular coagulation; GM-CSF, Granulocyte-monocyte colony-stimulating factor; HA, hemagglutinin; NA, neuraminidase; NET, neutrophil extracellular traps.

Influenza viruses are primarily transmitted *via* inhalation of infectious particles when an infected person coughs or sneezes. However, airborne and fomite transmission may also contribute to viral spreading (136). During infection HA binds to the sialic acid (SA)-terminated glycans present at the cell membrane. NA facilitates the release of virus progeny by cleaving SA residues from the cell surface (70) (**Figure 5**).They primarily target epithelial cells of the upper and lower respiratory tract, causing pneumonia, but also encephalopathy and myocarditis (142, 143). In severe cases pneumonia is often accompanied by acute lung injury (ALI) or even acute respiratory distress syndrome (ARDS), which is characterized by alveolar capillary damage, oedema, parenchymal haemorrhages, pulmonary microvascular thrombosis and hyperinflammatory cytokine responses (142, 144). ALI and ARDS also correlate with organ failure, ICU admission and a high fatality rate (145).

Severe influenza infections can also have major impact on the haemostatic system, with thrombosis and bleedings potentially occurring at the same time. On the one hand, influenza infections are often associated with thrombocytopenia and elevated mean platelet volume (MPV), indicating elevated thrombopoiesis, potentially due to elevated platelet activation and phagocytosis of viral particles (70, 145–148). Accordingly, pulmonary, alveolar and interstitial haemorrhages are frequent complications (70, 137, 149). On the other hand, influenza infection and associated platelet activation increases the risk of thrombus formation.

Influenza-stimulated platelets infiltrate the lungs of infected individuals (137, 149), where they form platelet-platelet and platelet-leukocyte aggregates (137, 147, 149), partially occluding blood vessels or small airways. In addition, neutrophil stimulation triggers NET release, which are highly cytotoxic and thus contribute to tissue damage and induce thrombosis. Interestingly, influenza-mediated platelet activation is not restricted to the lungs, as NET components and platelets co-localize in the heart of influenza-infected mice (144, 149). Also, enhanced deep vein thrombosis (DVT) and pulmonary thromboembolism may occur in influenza patients (142). These data indicate that thrombocytopenia and platelet activation contribute to influenza-associated pulmonary injuries caused by systemic inflammation and local leukocyte infiltrates, thereby fostering pulmonary thrombosis and haemorrhages (71, 147). In addition, in mice anti-platelet therapy [including aspirin, P2Y_12_ blockage and antagonists of α_IIb_β_3_ or protease-activated receptor 4 (PAR4)] reduces platelet aggregation, leukocyte recruitment and infiltration, viral reproduction and alveolar damage, ameliorating survival and underlining the contribution of platelets to influenza-mediated lung pathologies (137, 149, 150). However, the role of PAR4 remains unclear as one study found a protective effect of PAR4 inhibition on survival in influenza-challenged mice (137), whereas another study reported a detrimental effect of PAR4 deficiency (150).

Recent findings suggest that communication between platelets and neutrophils *via* CXCL4 is a prerequisite for viral removal and efficient immune response (151). However, dysregulated platelet-neutrophil crosstalk also contributes to influenza-mediated pathologies. Platelet derived complement factor C3 and GM-CSF are regarded as key proteins for platelet-neutrophil communication. Activated platelets form aggregates with neutrophils and secrete C3 from their granules which induces NET formation and thus helps to entrap and kill viruses. In turn, neutrophils trigger platelets to release GM-CSF, which serves as a negative feedback mechanism by reducing C3-mediated NET formation. Dysregulated platelet-neutrophil communication and surplus of C3 and NETs are predictors of acute myocardial infarction and myocardial infarct size and are therefore believed to increase the risk of influenza-associated myocardial infarct (62). Furthermore, platelet aggregation involving integrin α_IIb_ was recently found to play an important role for platelet-leukocyte interplay during influenza as mice deficient for α_IIb_ displayed reduced pulmonary neutrophil influx and NET formation upon challenge with influenza. Similarly, thrombin and PAR4 inhibition also diminished platelet accumulation, neutrophil influx and NET formation in the lungs and reduced pulmonary oedema without affecting viral load, underlining the importance of platelet aggregation for neutrophil-mediated tissue damage (152).

Moreover, platelets contribute to influenza-associated pathologies by interacting with infected endothelial cells. Both *in vitro* and *in vivo* platelets adhere to endothelial cells upon infection with influenza A subtypes H1N1 and H3N2, mediated by the interaction of endothelial fibronectin and platelet integrin α_5_β_1_. In influenza-infected mice anti-platelet treatment using aspirin thus blocks platelet adhesion to the endothelium, leading to reduced arterial hypoxemia and improved survival (95).

### SARS-CoV-2

SARS-CoV-2, the causative pathogen of coronavirus-induced disease 2019 (COVID-19), newly emerged in 2019 and led to an ongoing global pandemic.

The COVID-19 pandemic has forced the scientific community to face complex challenges in order to understand the molecular and cellular processes responsible for COVID-19 pathology and improve patient treatment. Although scientific advancements could build on knowledge of the closely related SARS-CoV-1 and Middle East respiratory syndrome coronavirus (MERS-CoV), SARS-CoV-2 shows distinct properties which require in-depth elucidation. Further, the rapid spread of SARS-CoV-2 demanded that new discoveries had to be reported at an unprecedented speed and made broadly available, often curtailing peer review processes and forcing researchers to lower their sights regarding tightly defined cohort composition. In particular, location and time of recruitment may influence clinical studies due to e.g. overtaxed healthcare systems and adapted treatment protocols. As a consequence, despite best efforts studies frequently yield contradictory results that will have to be verified in the future under controlled and comparable settings.

COVID-19 primarily affects the upper and lower respiratory tract, leading to respiratory distress or failure, however complications involving the haemostatic system such as thrombosis, thromboembolism and bleeding events are common, particularly in patients requiring intensive care unit (ICU) treatment (153–155). Additionally, platelet counts are reduced in COVID-19, hinting towards an involvement of primary haemostasis, though counts do not drop to the same extent as during non-COVID-19 pneumonia and clinical thrombocytopenia is rare (156–160). Of note, thrombocytopenia occurs more frequently in severe than non-severe infection (161) and is associated with significant bleeding complications (153).

Despite overall minor reduction of circulating platelets, COVID-19 is associated with increased levels of immature platelets, indicating enhanced platelet turnover (162). Blood smears of COVID-19 patients further revealed the presence of giant platelets, corroborating reports of increased MPV (47, 163, 164). Increased MPV is associated with disease severity (164, 165) and acute kidney failure in COVID-19 (166), but interestingly not with ICU requirement (48).

**Figure 6:**
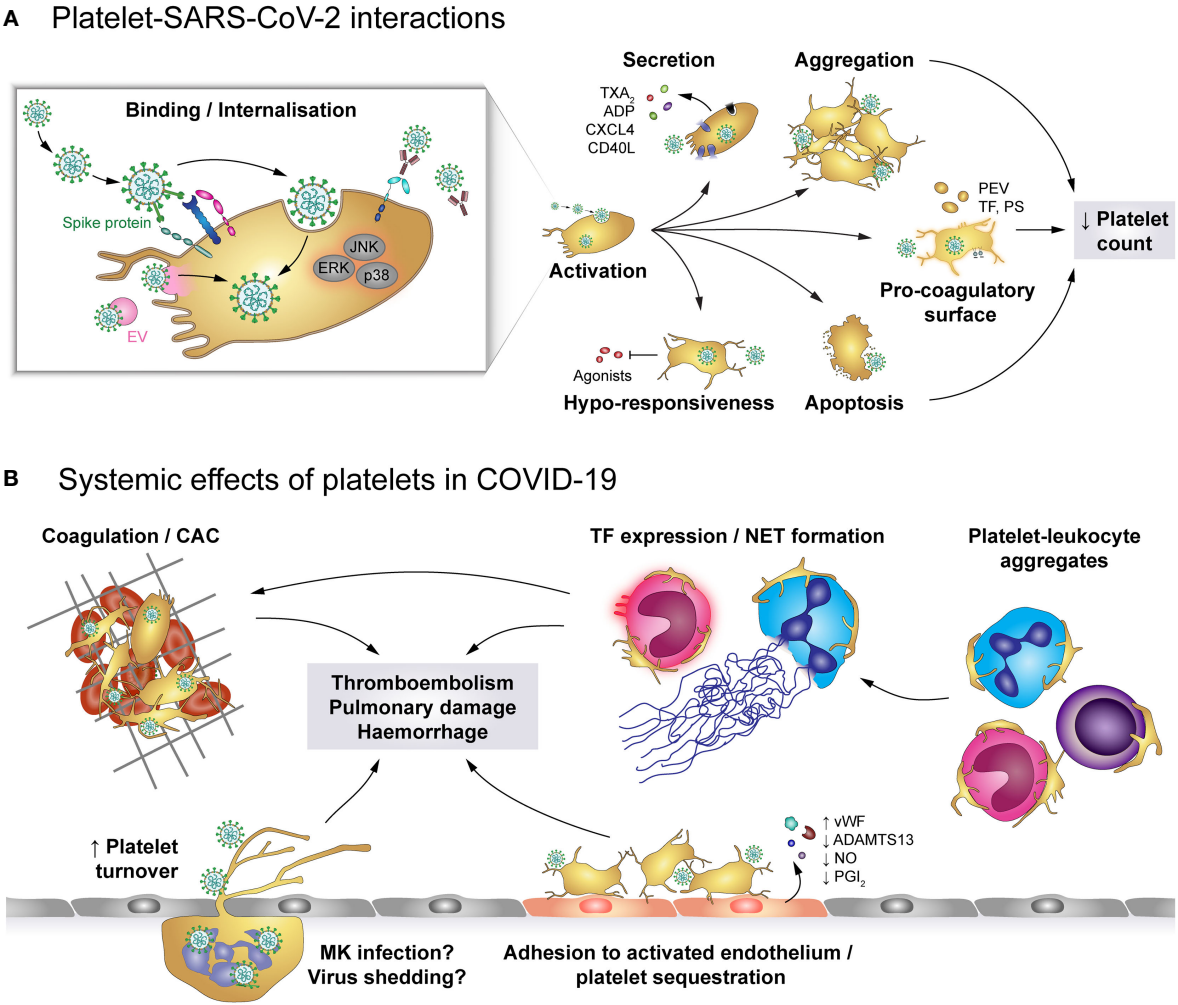
The role of platelets in COVID-19. **(A)** SARS-CoV-2 may enter platelets upon binding to ACE2/TMPRSS2 or EMMPRIN receptors or by hitchhiking on extracellular vesicles (EV) that fuse with the platelet plasma membrane, though SARS-CoV-2-containing immune complexes are also recognised by FcγRIIA. Binding and/or internalisation of SARS-CoV-2 stimulates platelet activation including degranulation and secretion, integrin activation and aggregation as well as exposure of pro-coagulant surfaces on platelets themselves or platelet-derived extracellular vesicles (PEV). Together with apoptosis of virus-bound platelets, these processes induce a reduction in circulating platelet count. Platelet hyper-activation in COVID-19 is accompanied by hypo-responsiveness to further stimulation. **(B)** Altered platelet behaviour in COVID-19 has systemic effects on pro-thrombotic and immunomodulatory platelet functions. Hyper-active and pro-coagulant platelets show enhanced adhesion to the inflamed endothelium and foster the development of COVID-19-associated coagulopathy (CAC). Accordingly, platelet turnover is increased in COVID-19. Formation of platelet-leukocyte aggregates triggers monocyte TF expression and NET formation, which add to the pro-coagulant microenvironment. In addition, infected megakaryocytes may shed virions to exacerbate infection. Together, these pathologic alterations increase the risk for thromboembolisms, pulmonary damage and haemorrhages. ACE2, Angiotensin-converting enzyme 2; ADP, adenosine diphosphate; ADAMTS13, A disintegrin and metalloproteinase with a thrombospondin type 1 motif, member 13; CAC, COVID-19-associated coagulopathy; CD40L, CD40 ligand; COVID-19, Coronavirus-induced disease 2019; CXCL4, chemokine (C-X-C motif) ligand 4; EMMPRIN, extracellular matrix metalloproteinase inducer; ERK, Extracellular signal-regulated kinase; FcγRIIA, Immunoglobulin γ Fc region receptor IIA; JNK, c-Jun N-terminal kinase; p38, p38 mitogen-activated protein kinase; MK, Megakaryocyte; EV, Extracellular vesicle; NET, neutrophil extracellular trap; PEV, Platelet-derived extracellular vesicle; NO, Nitric oxide; PGI_2_, Prostaglandin I_2_ (prostacyclin); PS, phosphatidylserine; SARS-CoV-2, Severe acute respiratory syndrome coronavirus 2; TF, Tissue factor; TMPRSS2, Transmembrane protease serine subtype 2; TXA_2_: thromboxane A_2_; vWF, von Willebrand factor.

The primary receptor for SARS-CoV-2 is ACE2 which binds to the viral spike protein and enables cell entry, facilitated by transmembrane protease serine subtype 2 (TMPRSS2) (**Figure 6**). Both ACE2 and TMPRSS2 were found to be expressed on human and mouse platelets and on the megakaryocytic cell line MEG-01 (47), mediating virus binding and internalisation (47, 158). Additionally, platelet EMMPRIN (CD147) is involved in spike protein-mediated platelet activation and SARS-CoV-2 may hitchhike on extracellular vesicles to enter platelets *via* membrane fusion, thereby circumventing the need for ACE2 (50, 63). While platelets do not seem to support viral replication, MEG-01 cells can be infected at least temporarily, leading to rising intracellular and shed virions which suggests successful replication (158). However, whether megakaryocyte infection also occurs *in vivo* and if it affects thrombopoiesis is currently unknown.

SARS-CoV-2 infection is associated with dysregulated platelet functions, affecting all phases of primary haemostasis. Platelets of COVID-19 patients express increased surface levels of CD62P and CD63 relative to either healthy controls or non-COVID-19 pneumonia patients (47, 48, 157, 167–170), demonstrating augmented basal platelet degranulation. In line, α-granule-contained PF4/CXCL4 and dense granule-contained serotonin (5-HT) are reduced within platelets, but increased in plasma (46), along with sCD40L, ADP and TXA_2_ (164, 167, 169). Of note, surface CD62P, CD63 and plasma TXA_2_ levels correlate with plasma fibrinogen, D-dimer and C-reactive protein, suggesting a link between enhanced basal platelet degranulation and the hyper-inflammatory and hyper-coagulatory milieu in COVID-19 (167). Additionally, platelets of COVID-19 patients also exhibit enhanced basal α_IIb_β_3_ activation relative to healthy donors or non-COVID-19 pneumonia patients (47, 168, 169).

*In vitro* and *in vivo* studies using a humanized mouse model demonstrate that SARS-CoV-2 virions dose-dependently enhance platelet degranulation, α_IIb_β_3_ activation, spreading, aggregation and thrombosis in a mechanism involving spike protein and platelet ACE2 (47), further underlining the link between SARS-CoV-2 infection and platelet hyper-activation. On the other hand, plasma of severely-ill COVID-19 patients also induces platelet CD62P and CD63 expression relative to control plasma (167), providing evidence that platelet activation in COVID-19 is also regulated by plasma components.

While the exact mechanisms of SARS-CoV-2-mediated platelet hyper-activation is still unclear, COVID-19 is associated with enhanced activation of phospholipase A_2_ (PLA_2_) and protein kinase Cδ (PKCδ) (46, 48), which are prominently involved in TXA_2_ generation and platelet degranulation, respectively. Furthermore, janus-activated kinase 3 (JAK3) and mitogen-activated protein kinases such as extracellular signal-regulated kinase (ERK), p38 and c-Jun-N-terminal kinase (JNK) are also triggered in COVID-19 or upon direct exposure to SARS-CoV-2 (47, 48), providing evidence of the complex and diverse dysregulation of the intracellular signalling network that may cause platelet hyper-activation and influence thrombotic risk. Indeed, in a murine thrombosis model SARS-CoV-2 spike protein enhances thrombosis only if mice were transfused with human ACE2-expressing platelets, but not in mice transfused with wildtype platelets (47).

Though reports on elevated basal platelet activation in COVID-19 appear very consistent, associations with disease severity are more variable with some studies showing higher platelet activation in more severe cases (47, 167), whereas other studies did not find any association between disease severity and platelet activation (46, 48).

Interestingly, despite evident platelet hyper-activation in COVID-19, effects on platelet responsiveness are unclear. In fact, initial studies reported facilitated agonist-induced CD62P expression, TXA_2_ release, adhesion, spreading and aggregation in COVID-19 relative to healthy controls (46, 48). However, accumulating evidence indicates that platelets of COVID-19 patients display a hypo-responsive phenotype that affects degranulation (168, 171), α_IIb_β_3_ activation (48, 157) and aggregation (172), potentially due to alterations in the proteome (173). Further, COVID-19 effects on platelet responsiveness were reported to differ between agonists (169) and to depend on agonist concentration (170) as well as disease stage (174). In line, *in vitro* thrombus formation under flow is decelerated during the early phase of disease, but comparable to healthy donors in intermediate and late stages, independently of platelet count or severity (175). This provides further evidence of the dynamic alterations of platelet function in COVID-19 and the difficulties in comparing studies of different sampling strategies.

Further, platelets of COVID-19 patients, especially of those with thrombosis, express higher levels of PS than control platelets (48, 176), but agonist-induced upregulation is impaired (177). Together with the occurrence of TF-positive platelets (178) and augmented circulating PMVs (46), these findings provide evidence of the pro-thrombotic and pro-coagulatory microenvironment in COVID-19, which results in thrombotic complications in lungs and other tissues (170). Though, in light of the observed hypo-responsiveness of platelets in aggravated COVID-19, the relative contributions of primary and secondary haemostasis to thromboembolic complications are still incompletely understood.

Hyper-activation of platelets in COVID-19 may not only contribute to elevated thrombotic risk, but could also mediate other disease symptoms such as thrombocytopenia. Aggravating disease severity is associated with increased levels of apoptotic platelets, which in turn negatively correlate with circulating platelet count. To distinguish apoptotic from pro-coagulant platelets, true apoptosis was corroborated by higher mitochondrial inner membrane potential and cytosolic calcium as well as augmented caspase 9 cleavage in addition to PS upregulation. Thereby, elevated platelet apoptosis was identified in COVID-19 patients requiring ICU treatment relative to both healthy controls and to COVID-19 patients in general ward. *In vitro* this pro-apoptotic phenotype can be reproduced by serum or IgGs derived from ICU patients, suggesting an FcγRIIA-mediated effect (176).

Apart from platelet apoptosis, declining circulating platelets could also be a result of platelet hyper-activation, leading to enhanced adhesion (46, 170) and thus potentially to sequestration. Indeed, platelet count negatively correlates with platelet degranulation markers (47) and associates with mortality (179) and bleeding risk (153, 180), but interestingly not with thrombosis (181). Platelet sequestration may be facilitated by activation of endothelial cells in COVID-19, which show impaired synthesis of nitric oxide and PGI_2_ (178). In combination with increased levels of vWF and decreased a disintegrin and metalloprotease with thrombospondin type motif 13 (ADAMTS13) in plasma (182), endothelial dysfunction thus generates a pro-thrombotic milieu that fosters the development of microthrombi and sequestration of hyper-active platelets.

Patients with COVID-19 also present with elevated levels of PLAs involving monocytes and neutrophils as well as CD4- and CD8-positive lymphocytes (48, 157, 167, 169). Elevated levels of platelet-monocyte aggregates (PMAs) were found predominantly in severe but not mild COVID-19 (167). Similarly, platelet-neutrophil aggregates (PNAs) also increase with disease severity and worsening blood oxygenation (170). Nevertheless, circulating PLAs are lower in non-survivors than survivors (174). Enhanced PLA formation in COVID-19 may of course be regulated by virus-induced leukocyte activation. However, platelet hyper-activation is also prominently involved as *in vitro* platelets from COVID-19 patients show higher PMA and PNA formation with naïve leukocytes than platelets from healthy donors (111, 170).

In line with platelet-mediated regulation of leukocyte function, monocytic TF expression in COVID-19 patients is higher on platelet-bound than on solitary monocytes (167) and patient-derived platelets promote NET formation over naïve platelets (170). These findings indicate that platelet activation may promote thromboembolic complications also *via* augmenting pro-coagulant leukocyte behaviour. Whether platelet hyper-activation and platelet responsiveness in COVID-19 also impact on immune responses and the ability to combat SARS-CoV-2 is currently still unexplored.

Overall, COVID-19 is clearly associated with platelet dysfunction, though its exact characteristics, dynamic changes and underlying mechanisms are still unclear. Current literature supports the idea that platelet dysfunction contributes to (micro)thrombotic events and may affect platelet counts, possibly even immune responses, which in turn could have repercussions for bleeding/thrombotic risk, organ function and ultimately survival.

Hence, the clinically-relevant effects of anti-platelet medication on COVID-19 morbidity and mortality are carefully investigated. Though, fitting to the controversial findings regarding platelet function in COVID-19, studies on the effect of anti-platelet medication also provide variable results.

While dual anti-platelet therapy improves hypoxemia (183) and aspirin has been associated with lowered risk for mechanical ventilation and ICU admission as well as decreased in-hospital mortality without affecting bleeding risk in some studies (184, 185), others found no protective effect of anti-platelet drugs against adverse thrombotic events, severity or mortality (186, 187). An ongoing large randomized controlled trial currently comprising almost 15.000 COVID-19 patients reports no impact of aspirin treatment on the risk for invasive ventilation or mortality (188), which fits to the hypo-reactive platelet phenotype described in various patient cohorts. Future studies will have to determine whether anti-platelet therapy might affect morbidity of COVID-19 patients by impairing other platelet-mediated processes, e.g. vascular surveillance or immunomodulation.

## Platelets and Viral Diseases – Treatment Options

Understanding the underlying mechanisms of platelet dysfunction in viral infectious disease and unravelling the consequences of their interplay with other cellular and non-cellular mediators represents a prerequisite to discover novel and safe therapeutic targets in this complex disease. Current anti-platelet strategies targeting COX-1 and/or P2Y_12_ to reduce platelet activation and the incidence of arterial thrombosis in patients with cardiovascular diseases. However, they dysregulate the haemostatic balance, leaving patients at risk of systemic side-effects such as bleeding complications.

During chronic and acute inflammatory diseases, including virus infections, distinct mechanisms of platelet activation occur, which are potentially insensitive to classical anti-platelet drugs, e.g. due to alternative pathways of platelet activation that may be of particular importance for immunothrombotic processes during severe infections. The use of thrombin inhibitors or thrombin receptor antagonists which target both primary and secondary haemostasis, represents another available approach, but also bears a risk for haemorrhages. Animal studies on influenza infections could show beneficial effects of the thrombin receptor PAR1 beyond inhibition of platelet activation (189). A recent study suggests that inflammatory and immune-thrombotic mechanisms of platelets can be diminished upon inhibition of PAR4 during influenza infection (152), leading to ameliorated survival (137). However, inhibition of hypo-responsive platelets by these classical platelet agonists might further increase the bleeding risk. Therefore, alternative strategies are necessary to dampen the risk of haemostatic dysregulations in viral diseases.

Recent studies focus on targeting primary platelet activation pathways e.g. *via* immunoreceptor tyrosine-based activation motif (ITAM)-containing collagen receptor GPVI/FcRγ-chain complex. While GPVI inhibition yielded encouraging results, reducing platelet aggregation without elevating major bleeding complications (190), nothing is yet known on potential beneficial effects in viral diseases. Also targeting immunoreceptor tyrosine-based inhibitory motif (ITIM)-containing receptors could provide an alternative approach for targeted platelet inhibition due to the role of these receptors in the downregulation of platelet ITAM-receptor signalling (191). Again, nothing is known yet on the effect of these inhibitors in viral diseases. However, as some viruses result in GPVI shedding and diminished GPVI responses, like all classical anti-platelet drugs also these inhibitors target platelets behind time. Interfering with platelet-virus interactions would therefore provide another interesting target, though this is likely to also affect immune-defence mechanisms and therefore accelerate the risk of unfavourable outcome.

Another crucial aspect is blocking immune-complex mediated platelet activation *via* FcγRIIA. The physiologic relevance of platelet FcγRIIA, which may facilitate both direct antimicrobial function of platelets as well as crosstalk with other immune cells, is currently unclear (192). This vicious interaction of immune complexes and platelet FcγRIIA is not only responsible for platelet activation in viral infections but also in a plethora of other diseases – including heparin-induced thrombocytopenia (HIT), autoimmune diseases like systemic lupus erythematosus (SLE) (193) or the more recently discovered vaccine-induced immune thrombotic thrombocytopenia (VITT). To date, no data exist on potential effects of drugs interfering with FcγRIIA signalling in viral infections. Investigations are complicated by the fact that mice do not express FcγRIIA on platelets. However, humanized mouse models do exist and for some viruses a crucial involvement of FcγRIIA for platelet activation and subsequent thrombocytopenia could be unravelled (193). Further studies are warranted to fully understand the impact of platelet inhibitors on viral infections. Beneficial effects of such interventions will likely depend on the virus type, disease stage and co-morbidities of patients. Therefore, experimental models and patient cohorts have to be carefully selected and results cautiously interpreted. Patients are often enrolled at different disease stages and not longitudinally monitored. Moreover, they often suffer from co-infections and/or other co-morbidities, which have to be considered. While some of these obstacles can be overcome by animal models, for some pathogens no appropriate models exist. Also, haemostatic processes as well as platelet surface receptor patterns significantly differ between mice and humans. As pandemics and concomitant haemostatic dysregulations will remain a recurrent threat, understanding the role of platelets in viral infections represents a timely and pivotal challenge.

## Author Contributions

Conceptualization: AA; Visualization: WS, AA; Funding Acquisition: WS, AA; All authors contributed to Writing, Original Draft Preparation and Writing, Review and Editing. All authors contributed to the article and approved the submitted version.

## Funding

This work is financially supported by grants of the Austrian National Bank to WS (OENB18450) and of the Austrian Federal Ministry of Education, Science and Research, the Medical-Scientific Fund of the Mayor of Vienna (COVID024) and the Austrian Science Fund (P32064 and P34783) to AA.

## Conflict of Interest

The authors declare that the research was conducted in the absence of any commercial or financial relationships that could be construed as a potential conflict of interest.

## Publisher’s Note

All claims expressed in this article are solely those of the authors and do not necessarily represent those of their affiliated organizations, or those of the publisher, the editors and the reviewers. Any product that may be evaluated in this article, or claim that may be made by its manufacturer, is not guaranteed or endorsed by the publisher.
